# Enhanced Recovery for Patients Undergoing Metabolic Bariatric Surgery in a Community Hospital: A Retrospective Pre–Post Quality Improvement Study

**DOI:** 10.1007/s11695-026-08846-4

**Published:** 2026-07-22

**Authors:** Rachel Trotter, Linda Shanks, Howie Brown, Anne M Miranda

**Affiliations:** 1 Department of Anesthesia and Bariatric Surgery, Mercy Health - Lorain Hospital, Lorain, USA; 2 Department of Nursing, University of Akron, Akron, USA

**Keywords:** Enhanced Recovery After Surgery (ERAS), Metabolic Bariatric Surgery, Gastric Bypass, Opioid Consumption, Postoperative Nausea and Vomiting, Quality Improvement, Community Hospital, Multimodal Analgesia

## Abstract

**Background:**

A standardized, multimodal Enhanced Recovery After Surgery (ERAS) protocol was implemented to improve postoperative outcomes in patients undergoing metabolic bariatric surgery (MBS). This quality improvement initiative evaluated the impact of a standardized, multimodal ERAS protocol on postoperative outcomes in MBS patients. Implemented at a community hospital where perioperative practices were inconsistent, this evidence-based pathway introduced a structured approach to perioperative care to enhance recovery and promote safer, more efficient outcomes.

**Methods:**

A pre- and post-quality improvement study was conducted among adults undergoing MBS at a Midwestern community hospital following implementation of a structured ERAS protocol. Patients younger than 18 years and those with long-term opioid use were excluded. The Model for Evidence-Based Practice Change guided protocol development and implementation. Pre-implementation and post-implementation cohorts were compared across primary endpoints, including PONV incidence, opioid consumption, and length of stay (LOS). Mann–Whitney U tests, and chi-square or Fisher’s exact tests were used (*p* < .05).

**Results:**

ERAS implementation resulted in a significant reduction in postoperative opioid use (Mdn = 22.5 vs. 80.0 MME; U = 143.50, *p* < .001). No significant differences were observed in PONV or LOS between groups. Protocol adherence was high (89.4%).

**Conclusion:**

A structured, multimodal ERAS protocol was an effective quality improvement strategy for patients undergoing MBS at a community hospital. Standardizing perioperative care was associated with a significant reduction in opioid requirements without increasing PONV or LOS, supporting broader adoption of ERAS pathways in bariatric surgery programs.

## Introduction

Obesity remains a major global health concern, with significant associated morbidity, mortality, and healthcare costs [1]. Metabolic bariatric surgery (MBS) is the most effective treatment for severe obesity and its associated comorbidities, with demonstrated long-term efficacy and safety [2]. Despite improvements in surgical technique, postoperative challenges such as postoperative nausea and vomiting (PONV), pain, and prolonged recovery remain clinically significant [3–5].

Enhanced Recovery After Surgery (ERAS) protocols are designed to improve perioperative outcomes by standardizing evidence-based practices across the surgical continuum [6–8]. In bariatric populations, these protocols target key drivers of delayed recovery, particularly PONV and pain [3, 9]. ERAS protocols incorporate multimodal, opioid-sparing analgesia, optimized antiemetic regimens, and regional anesthesia techniques to reduce perioperative opioid exposure while improving symptom control [9, 10]. Patients undergoing MBS are at increased risk for opioid-related adverse events, including respiratory complications and persistent opioid use, further emphasizing the need to minimize opioid exposure in this population [11, 12]. Given the ongoing opioid epidemic, reducing perioperative opioid use remains a critical strategy to improve patient safety and postoperative outcomes [11]. Improved control of nausea facilitates earlier tolerance of oral intake, a key determinant of discharge readiness and hospital LOS [4, 5]. Although prior studies have demonstrated favorable outcomes following ERAS implementation, adoption of ERAS protocols in MBS remains variable, and data describing real-world implementation in community hospital settings are limited [7–9]. As bariatric programs pursue quality benchmarks such as the Metabolic and Bariatric Surgery Accreditation and Quality Improvement Program (MBSAQIP), the need for standardized, evidence-based perioperative pathways becomes increasingly important [13]. In response, this study evaluates the clinical impact and feasibility of a standardized ERAS protocol in a community-based practice environment.

## Purpose

At the project site, inconsistent perioperative practices led to variability in postoperative outcomes among patients undergoing MBS, underscoring the need for an evidence-based ERAS protocol. The purpose of this evidence-based quality improvement project was to implement a multimodal ERAS protocol to standardize perioperative care by reducing PONV, opioid use, and LOS.

## Patients and Methods

### Study Design and Setting

This retrospective pre-post quality improvement study was conducted at a Midwestern community-based, short-term acute care hospital with 196 beds and 12 operating rooms. The hospital performs robotic-assisted laparoscopic metabolic and bariatric surgery (MBS) and was pursuing MBSAQIP accreditation during the study period. An enhanced recovery after surgery (ERAS) protocol was implemented on August 1, 2025. Outcomes were compared between patients who underwent MBS during the pre-implementation period (March–July 2025) and those treated during the three-month post-implementation period (August 1–October 31, 2025). A total of 59 patients were included: 30 in the pre-implementation group and 29 in the ERAS group.

### Patient Population

Adult patients undergoing primary laparoscopic or robotic Roux-en-Y gastric bypass (RYGB) or sleeve gastrectomy were eligible for inclusion. Patients younger than 18 years and those with chronic pain requiring long-term opioid therapy were excluded. All procedures were performed using a minimally invasive robotic-assisted laparoscopic approach. Concurrent hiatal hernia repair was performed when clinically indicated. For statistical analyses, patients were categorized according to the primary bariatric procedure.

### ERAS Protocol

The Model for Evidence-Based Practice Change was used to guide the development and implementation of a standardized ERAS protocol for patients undergoing MBS [14]. The process included identifying practice variability, engaging stakeholders, reviewing evidence-based practice, and developing, implementing, and evaluating the protocol. The ERAS protocol was implemented on August 1, 2025.

**Fig. 1:**
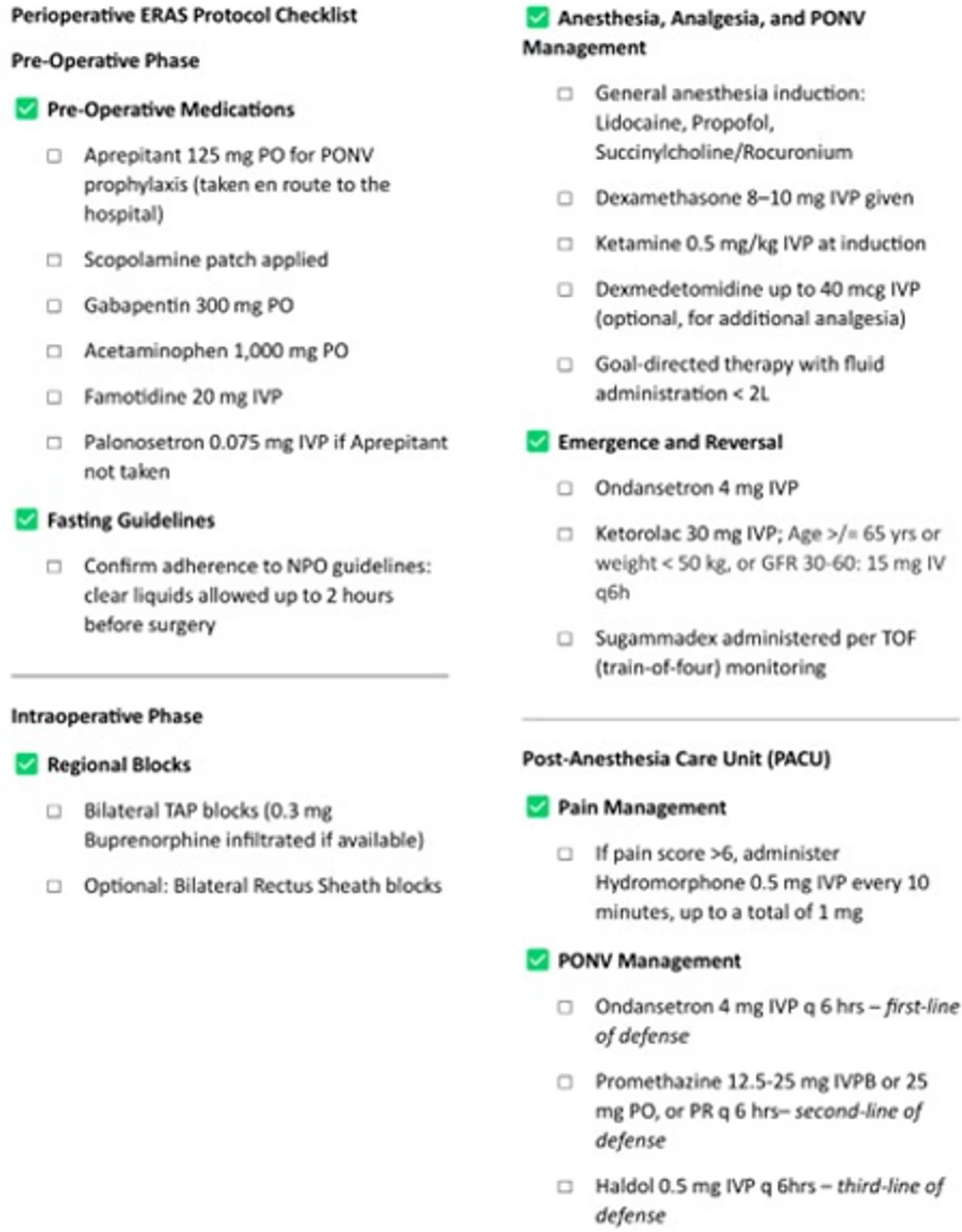
Perioperative ERAS protocol checklist outlining preoperative, intraoperative, and postoperative care components. Abbreviations: ERAS, Enhanced Recovery After Surgery; PONV, postoperative nausea and vomiting; PACU, post-anesthesia care unit; TAP, transversus abdominis plane

The intervention consisted of a standardized multimodal ERAS protocol incorporating preoperative, intraoperative, and postoperative components. Preoperative elements included patient education, shorter fasting intervals, and multimodal pharmacologic prophylaxis for PONV and pain. Intraoperative management emphasized opioid-sparing anesthesia through multimodal analgesia, prophylactic antiemetic administration, regional anesthesia techniques, and goal-directed fluid therapy. Postoperative care focused on standardized pain management, rescue antiemetic therapy, early mobilization and oral fluid intake, and discharge according to institutional bariatric surgery criteria. A detailed perioperative ERAS checklist was developed to facilitate standardized implementation across phases of care and is shown in Fig. 1.

### Data Collection and Outcome Measures

Data were collected through retrospective review of the electronic medical record using de-identified patient data. Variables included demographic characteristics, comorbidities, American Society of Anesthesiologists (ASA) classification, surgical characteristics, postoperative antiemetic administration, opioid consumption, hospital LOS, and ERAS protocol adherence.

The primary outcomes were PONV, postoperative opioid consumption, and hospital LOS. Postoperative nausea and vomiting were operationally defined as the administration of rescue antiemetic medication in either the post-anesthesia care unit (PACU) or inpatient setting. Opioid consumption was measured as cumulative morphine milligram equivalents (MME) administered during hospitalization and served as a surrogate measure of postoperative pain management. Length of stay was defined as the number of hours from hospital admission to discharge. Discharge readiness was determined according to institutional bariatric surgery criteria and included tolerance of oral fluids, adequate control of pain and nausea with oral medications, return to baseline mobility and respiratory status, successful voiding, and surgeon approval for discharge.

Protocol adherence was evaluated in the ERAS cohort and calculated as the proportion of required protocol components received by each patient. Adherence was assessed using a standardized perioperative checklist designed to support consistent implementation across phases of care.

### Statistical Analysis

Although this project was conducted as a quality improvement initiative and enrolled all eligible patients during the defined study period, an a priori power analysis was performed using total MME consumption data reported by Ma et al. (2020) to estimate the required sample size. The analysis indicated that with 80% power and a two-tailed α of 0.05, 23 patients per group would be required to detect a difference in total MME. To account for potential attrition, oversampling was performed by enrolling an additional 15% of patients. The final sample in this quality improvement project included 29 patients in the ERAS group and 30 patients in the control group, exceeding the minimum sample size estimated by the power analysis.

Continuous variables were assessed for normality using the Shapiro–Wilk test. Independent t-tests were used to compare normally distributed continuous variables, whereas Mann–Whitney U tests were used for nonparametric comparisons. Categorical variables were analyzed using chi-square or Fisher’s exact tests, as appropriate. Statistical significance was established at *p* <.05.

### Ethical Considerations

This project was reviewed by the appropriate institutional review boards and determined to be exempt as an evidence-based quality improvement initiative. Because the study involved retrospective analysis of de-identified data, informed consent was waived. This exemption was based on the project’s quality improvement nature and the retrospective use of de-identified data.

## Results

### Participant Characteristics

A total of 59 patients were included in the analysis, with 29 patients in the ERAS group and 30 patients in the pre-implementation group. The mean age of the overall sample was 46.49 years (SD = 12.73), and the mean body mass index (BMI) was 46.52 kg/m² (SD = 8.25). Age was normally distributed and comparable between groups. BMI was not normally distributed (Shapiro–Wilk *p* =.006); however, no significant differences were observed between groups.

Categorical variables, including sex, ASA classification, and associated comorbidities (diabetes mellitus, hypertension, obstructive sleep apnea, hyperlipidemia, gastroesophageal reflux disease, and surgical procedure), were similar between groups (all *p* >.05). Because expected cell counts were low, ASA classification was not compared statistically, although most patients in both groups were classified as ASA III.

The distribution of MBS was also comparable between groups (χ² (2, *N* = 59) = 0.67, *p* =.716). Roux-en-Y gastric bypass was the predominant procedure in both groups (93.1% vs. 86.7%), with a small number of sleeve gastrectomies and conversion procedures performed. Baseline demographic, clinical, and surgical characteristics are summarized in Table 1.

**Table 1:** Demographic and baseline clinical characteristics of participants (*N* = 59). Values are presented as mean (SD), n (%), or percentage unless otherwise indicated. BMI = body mass index; GERD = gastroesophageal reflux disease; ASA = American Society of Anesthesiologists; ns = not statistically significant

Variable	Total Sample	ERAS (*n* = 29)	Control (*n* = 30)	Test Statistic
Age (years)	*M* = 46.49 (SD = 12.73)	*M* = 45.34 (SD = 13.07)	*M* = 47.60 (SD = 12.51)	ns
BMI (kg/m²)	*M* = 46.52 (SD = 8.25)	*M* = 46.38 (SD = 7.81)	*M* = 46.66 (SD = 8.80)	Shapiro–Wilk *p* =.006; ns
Female (%)	83.1%			χ²(1) = 0.57, *p* =.451
Diabetes mellitus (%)	39.0%			χ² = 2.07, *p* =.150
Hypertension (%)	61.0%			χ² = 0.82, *p* =.365
Obstructive sleep apnea (%)	94.9%			χ² = 0.32, *p* =.574
Hyperlipidemia (%)	23.7%			χ² = 0.29, *p* =.590
Gastroesophageal reflux (%)	52.5%			χ² = 0.02, *p* =.902
Procedure (type)				χ² = 0.67, *p*=.716
Roux-en-Y gastric bypass (%)	53 (89.8) %	27 (93.1) %	26 (86.7) %	
Sleeve gastrectomy (%)	3 (5.1) %	1 (3.4) %	2 (6.7) %	
Conversion sleeve to Roux-en-Y gastric bypass (%)	3 (5.1) %	1 (3.4) %	2 (6.7) %	

### Postoperative Nausea and Vomiting

Overall, 21 patients (35.6%) required at least one antiemetic, whereas 38 patients (64.4%) required none. There was no significant association between ERAS implementation and postoperative antiemetic use (χ²(1, *N* = 59) = 0.52, *p* =.472). Due to non-normal distribution and low expected counts, Mann–Whitney U tests were performed for antiemetic administration. There was no difference in PACU antiemetic use between groups (mean rank 30.00 vs. 30.00; *U* = 435.00, *p* = 1.00). Floor antiemetic use showed a non-significant trend toward reduction in the ERAS group (mean rank 28.62 vs. 31.33; *U* = 395.00, *Z* = − 0.75, *p* =.454).

### Postoperative Opioid Consumption

**Fig. 2:**
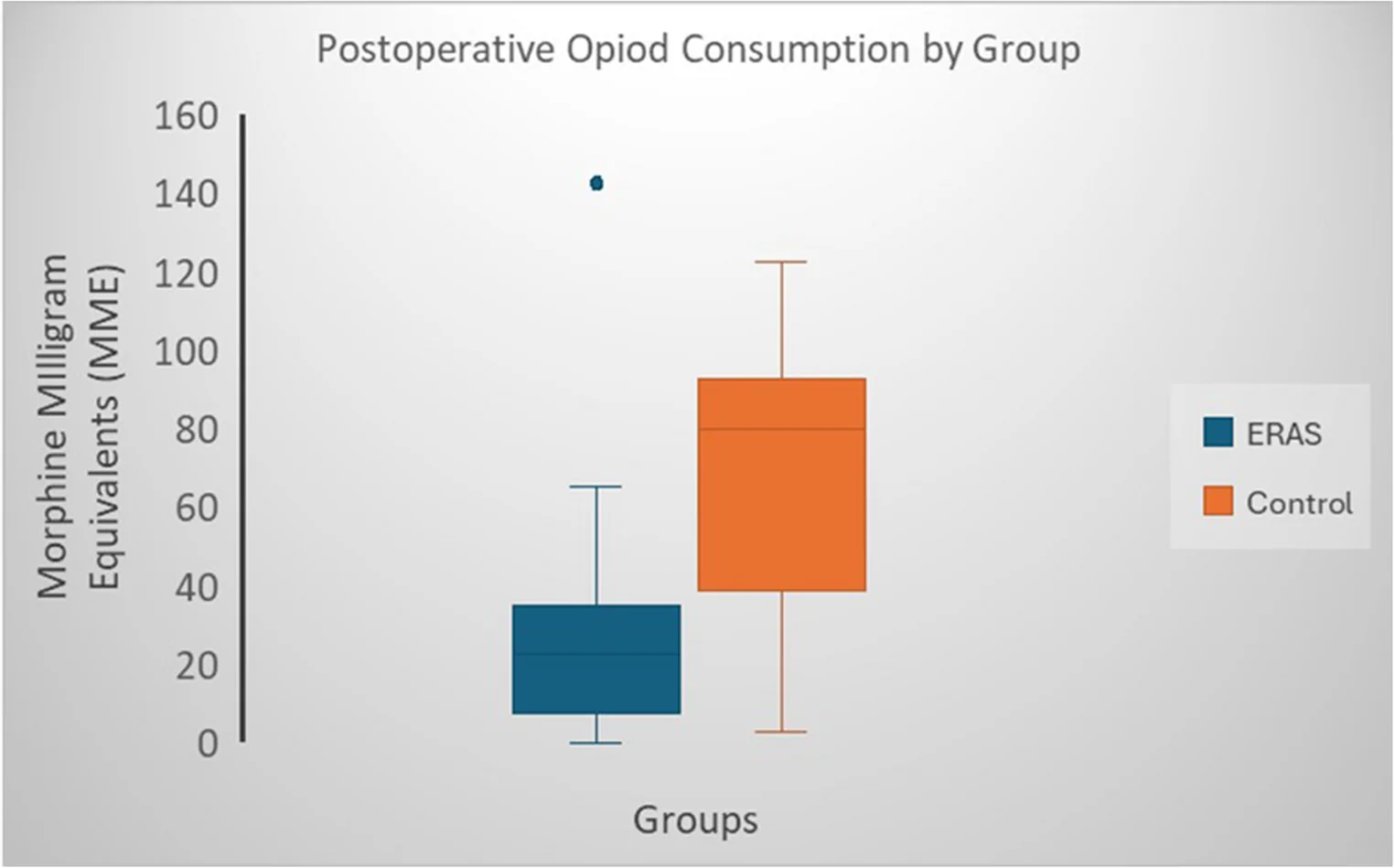
Postoperative opioid consumption (morphine milligram equivalents, MME) by group. The boxplot displays the median, interquartile range, and distribution of opioid use for ERAS and control groups. Outliers are represented by individual data points

Postoperative opioid use differed significantly between groups. Patients in the ERAS group required substantially fewer opioids compared with the control group (*Mdn* 22.5 vs. 80.0 MME), representing a clinically meaningful reduction. This difference was statistically significant (*U* = 143.50, *Z* = − 4.42, *p* <.001). The distribution of opioid consumption is illustrated in Fig. 2, demonstrating a lower median and narrower interquartile range in the ERAS group.

### Length of Stay

**Fig. 3:**
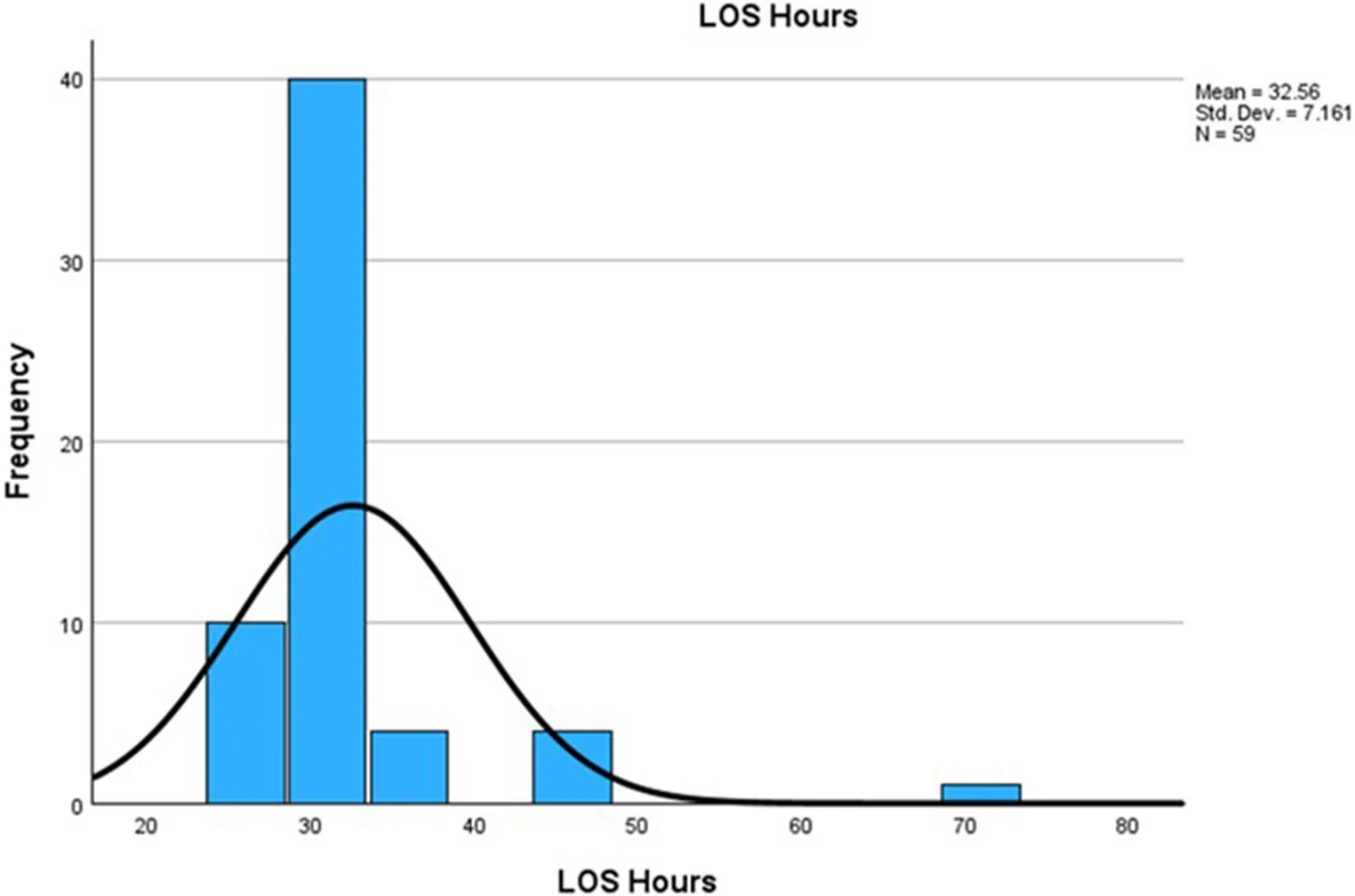
Distribution of LOS in hours for the overall study sample. The histogram illustrates the frequency distribution of LOS values and shows a non-normal distribution with an outlier

Length of stay was not normally distributed and included an outlier. Median LOS was slightly lower in the ERAS group (31.0 h) compared with the control group (31.5 h); however, this difference was not statistically significant (*U* = 337.00, *Z* = − 1.50, *p* =.134). Figure 3 presents the LOS distribution for the overall sample.

### ERAS Protocol Adherence

Adherence to ERAS protocol components was high. Scopolamine, gabapentin, acetaminophen, and bilateral transversus abdominis plane (TAP) blocks were administered in 100% of patients. Dexamethasone and ondansetron were administered in 96.6% of patients, and famotidine in 93.1%. Ketamine adherence was 79.3%, whereas ketorolac (65.5%) and aprepitant or palonosetron (65.0%) demonstrated the lowest adherence rates.

Ketorolac was withheld in patients with a documented allergy to nonsteroidal anti-inflammatory drugs (NSAIDs), and dose adjustments were made for patients aged > 65 years, with body weight < 50 kg, or with a glomerular filtration rate (GFR) of 30–60 mL/min/1.73 m². At the outset of protocol implementation, preoperative nursing documentation did not consistently indicate whether aprepitant had been administered before hospital arrival. As a result, a standardized documentation field was subsequently introduced, improving compliance. Some patients during the early implementation period may have received aprepitant at home without corresponding documentation in the medical record.

**Fig. 4:**
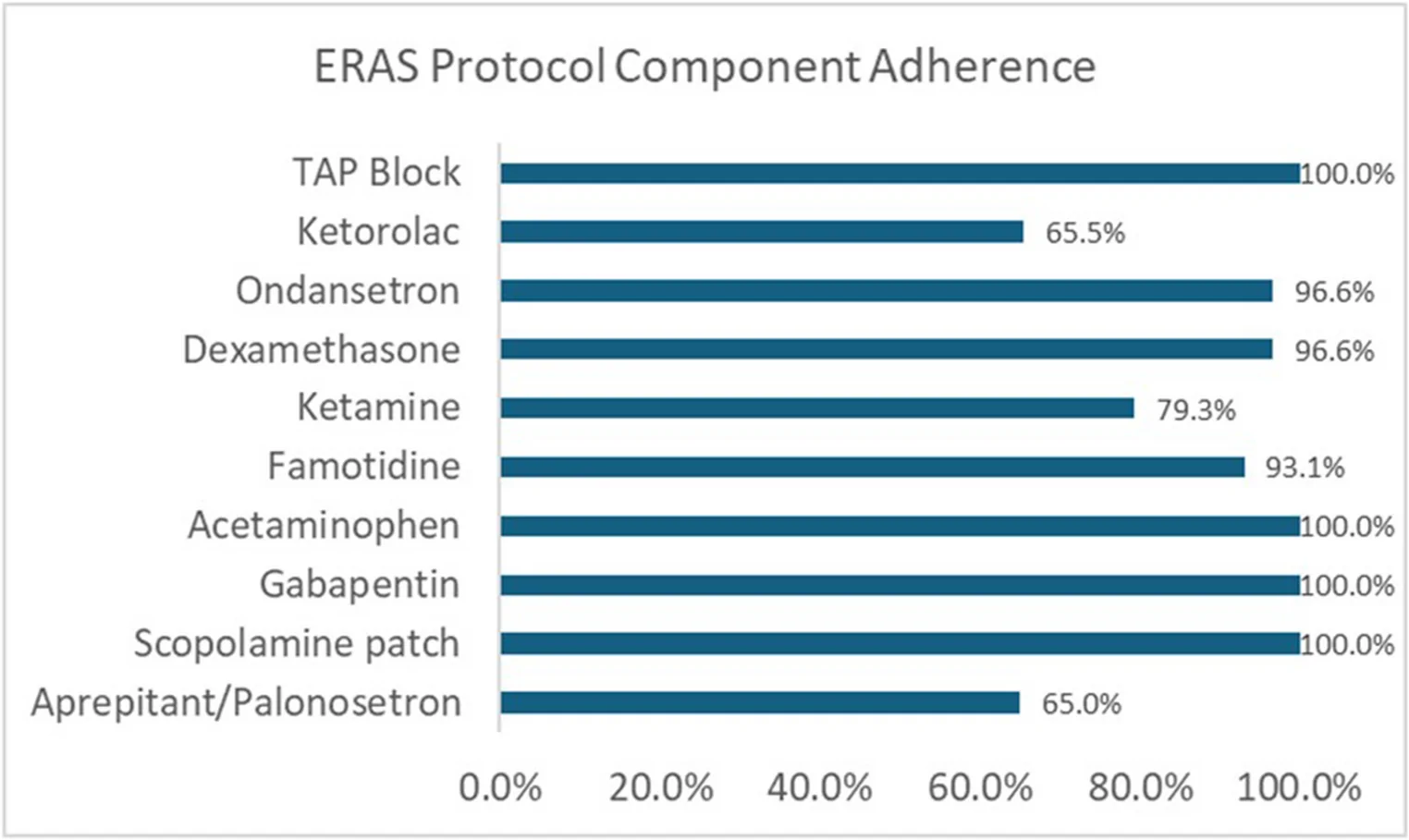
ERAS protocol component adherence among intervention patients. Adherence reflects the proportion of patients receiving each required protocol component. Dose variations were considered adherent, whereas clinically appropriate omissions were classified as nonadherent. Palonosetron was considered adherent only when aprepitant was not administered. Dexmedetomidine, buprenorphine infiltration, rectus sheath block, and intravenous fluids were excluded from adherence scoring

Overall, adherence to the ERAS protocol was 89.4%, corresponding to a mean of approximately 9 of 10 components received per patient. Adherence was defined as the proportion of patients receiving each required component. Dose variations were considered adherent, whereas clinically appropriate omissions were classified as nonadherent. Palonosetron was considered adherent only when aprepitant was not administered. Dexmedetomidine, buprenorphine infiltration, rectus sheath block, and intravenous fluids were excluded from adherence scoring. Component-level adherence is presented in Fig. 4.

## Discussion

Implementation of a standardized ERAS protocol for MBS was associated with a significant reduction in postoperative opioid consumption. Median opioid use decreased from 80.0 MME in the control group to 22.5 MME in the ERAS group, representing a clinically meaningful reduction in opioid exposure. These findings support the effectiveness of multimodal, opioid-sparing analgesic strategies and are consistent with previous bariatric ERAS studies demonstrating reduced postoperative opioid requirements [5, 8, 10, 15–21].

Minimizing opioid exposure is especially important for patients with obesity, who face increased risks of opioid-related respiratory complications and persistent postoperative opioid use [8, 10–12, 19, 21, 22]. In the present study, standardized postoperative pain scores were not available for retrospective review. Consequently, opioid consumption was used as a proxy measure of pain management, which may not fully reflect patient-reported pain experiences despite providing an objective measure of analgesic requirements.

No statistically significant differences were observed in PONV or LOS, although trends favored improvement. Because multiple patient, surgical, and anesthesia-related factors can influence PONV, the relatively small sample size may have limited detection of differences. In addition, defining PONV by rescue antiemetic use may have missed symptoms that were unreported or did not require medication. Similarly, LOS may have been influenced by institutional discharge processes and logistical factors unrelated to clinical recovery, including the timing of discharge orders, nursing workflows, transportation availability, and surgeon approval for discharge. Although reductions in LOS have been reported following ERAS implementation, smaller studies and early implementation phases may not demonstrate significant improvements [7–9].

High protocol adherence contributed to the observed reduction in opioid use and is consistent with prior literature demonstrating improved outcomes with greater ERAS compliance [5–8, 22]. The use of a standardized perioperative checklist supported consistent implementation across phases of care [6]. Successful implementation required strong interprofessional collaboration among anesthesia providers, surgeons, nursing staff, and pharmacy teams. This approach aligns with established Models for Evidence-Based Practice Change and supports coordinated delivery of perioperative care [14]. Implementation of ERAS protocols also aligns with MBSAQIP quality initiatives, supporting standardized care in community hospital settings [13].

### Limitations

This study has several limitations. The retrospective pre-post design limits the ability to conclude that the ERAS protocol alone caused the observed improvements, as other factors may have contributed to the results. The study was conducted at a single community hospital and involved a single bariatric surgeon, which may limit generalizability. Although the sample size exceeded the minimum requirement identified through an a priori power analysis for opioid consumption, the study may have been underpowered to detect differences in the PONV and LOS outcomes.

The evaluation period was limited to the first three months following implementation and may not reflect long-term adherence or sustainability. Standardized postoperative pain scores were not available for retrospective review; therefore, opioid consumption was used as a surrogate measure of pain management and may not fully reflect patient-reported pain experiences. Similarly, PONV was defined as the administration of a rescue antiemetic, which may underestimate symptoms that were not reported or treated. Finally, the study population was predominantly patients undergoing Roux-en-Y gastric bypass, limiting its applicability to other bariatric procedures.

## Conclusion

Implementation of a standardized ERAS protocol for metabolic bariatric surgery was feasible in a community hospital setting and was associated with a significant reduction in postoperative opioid consumption. Although PONV and LOS were not significantly reduced, trends favored improvement. These findings support the use of standardized ERAS pathways to promote opioid-sparing perioperative care, enhance recovery, and advance adherence to MBSAQIP quality standards. Further research with larger sample sizes and longer follow-up periods is warranted to evaluate the impact of ERAS implementation on additional postoperative outcomes.

## Data Availability

The datasets generated and/or analyzed during the current study are not publicly available due to institutional privacy policies and the use of protected health information but may be available from the corresponding author on reasonable request and with permission from Mercy Health – Lorain Hospital.
